# The Effect of Cyanobacterial LPS Antagonist (CyP) on Cytokines and Micro-RNA Expression Induced by *Porphyromonas gingivalis* LPS

**DOI:** 10.3390/toxins10070290

**Published:** 2018-07-16

**Authors:** Monica Molteni, Annalisa Bosi, Carlo Rossetti

**Affiliations:** Laboratorio di Biologia Applicata, Dipartimento di Medicina e Chirurgia, Università degli Studi dell’Insubria, Via Dunant, 3-21100 Varese, Italy; bosi.annalisa@gmail.com (A.B.); carlo.rossetti@uninsubria.it (C.R.)

**Keywords:** LPS, *Porphyromonas gingivalis*, *Oscillatoria planktothrix* FP1, TNF-α, IL-1β, IL-8, miRNA

## Abstract

Lipopolysaccharide (LPS) from *Porphyromonas gingivalis* (Pg-LPS) is a key bacterial structure involved in the maintenance of a chronic pro-inflammatory environment during periodontitis. Similar to other gram-negative LPS, Pg-LPS induces the release of pro-inflammatory cytokines through interaction with Toll-Like Receptor 4 (TLR4) and is able to stimulate negative TLR4 regulatory pathways, such as those involving microRNA (miRNA). In this work, we employed CyP, an LPS with TLR4-MD2 antagonist activity obtained from the cyanobacterium *Oscillatoria planktothrix FP1*, to study the effects on pro-inflammatory cytokine production and miRNA expression in human monocytic THP-1 cells stimulated with Pg-LPS or *E. coli* LPS (Ec-LPS). Results showed that CyP inhibited TNF-α, IL-1β and IL-8 expression more efficiently when co-incubated with Pg-LPS rather than with Ec-LPS. The inhibition of pro-inflammatory cytokine production was maintained even when CyP was added 2 h after LPS. The analysis of the effects of CyP on miRNA expression showed that, although being an antagonist, CyP did not inhibit miR-146a induced by Pg-LPS or Ec-LPS, whereas it significantly inhibited miR-155 only in the cultures stimulated with Ec-LPS. These results suggest that CyP may modulate the pro-inflammatory response induced by Pg-LPS, not only by blocking TLR4-MD2 complex, but also by preserving miR-146a expression.

## 1. Introduction

*Phorphyromonas gingivalis* (*P. gingivalis*) is a Gram-negative bacterium implicated in periodontitis, a chronic disease characterized by aggressive immune and inflammatory activation in the oral connective tissue, leading to an irreversible destruction of the tissue supporting the teeth and of the alveolar bone [1]. The chronic presence of periodontopathic bacteria infecting gingival tissue has also been associated with other systemic diseases, such as cardiovascular diseases [2]. The mechanisms by which *P. gingivalis* can efficiently colonize gingival tissues and evade host immune response are currently unknown. Lipopolysaccharide (LPS) expressed on the cell wall of *P. gingivalis* (Pg-LPS) is a well-known bacterial component that plays a major role in the release of pro-inflammatory mediators by immune cells which reside in the oral mucosa or that are recruited during infection [3,4]. It has been hypothesized that *P. gingivalis* may be able to modify LPS structures expressed on the bacterial surface in order to escape immunological control [5,6]. However, once infection is well established, *P. gingivalis* directly contributes, with other periodontopathic bacteria, to establish a high pro-inflammatory environment in which polymorphonuclear leukocytes and macrophages (stimulated by LPS and by other bacterial products) secrete high amounts of pro-inflammatory cytokines that cause bone resorption [7]. Anti-bacterial agents are not completely effective in the eradication of periodontal infection. Moreover, bacterial killing does not eliminate LPS, which is stable and can persist in the gingival environment [7].

Differently from *E. coli* classical LPS structure, which is mainly characterized by the presence of bi-phosphorylated, hexa-acylated lipid A [8], *P. gingivalis* can produce heterogeneous LPS composed of monophosphorylated tetra- and penta-acylated lipid A structures, showing different pro-inflammatory stimulatory activities [5,6,9,10]. LPS molecules obtained from gram-negative bacteria can induce a pro-inflammatory response by innate immune cells which is mediated by the interaction with the specific Pattern Recognition Receptor (PRR), Toll-Like Receptor 4 (TLR4) [11]. Pg-LPS does not represent an exception: although it was initially hypothesized that Pg-LPS could interact also with TLR2 [9], several experimental approaches using either synthetic or natural ultra-purified Pg-LPS have definitively excluded this possibility [12,13,14,15]. The variability of the lipid A moiety in the LPS structures of Gram-negative bacteria significantly contributes to the intensity of the pro-inflammatory response induced, depending mainly on the lipid A acylation and phosphorylation pattern [16]. Commercially available Pg-LPS preparations have been shown to contain, as a major component, the penta-acylated form of lipid A in their LPS structure [15]. This lipid A has been demonstrated to induce TLR4-mediated NF-κB signaling activation with a significant up-regulation of IL-6 and IL-8 production by human gingival fibroblasts [10,17]. Similar to LPS from other Gram-negative bacteria, Pg-LPS has been shown to induce negative TLR4 regulatory pathways, such as those involving microRNA (miRNA) [18]. In response to enterobacterial LPS, changes in the expression of selected miRNA, namely miR146a, miR-146b, miR-155, and miR-21 have been identified [19,20,21]. Some papers have reported significant induction of miR-146a, miR-155 in cell cultures of human gingival fibroblast and monocytic cell lines stimulated with Pg-LPS [22,23]. 

LPS represents an important component of the outer membrane of cyanobacteria [24]. Recently, we characterized the biological activity of CyP, which is a LPS structure extracted from the freshwater cyanobacterium *Oscillatoria planktothrix* sp. *FP1*. CyP is composed of a polysaccharide chain mainly consisting of 3-substituted α-L-rhamnose units in the O-antigen region, by a core region rich in galacturonic acid, and by non-phosphorylated lipid A with two to four acyl chains [24,25,26]. Differently from Gram-negative LPS, CyP did not induce any pro-inflammatory response in monocytes, macrophages, or dendritic cells [27,28]. Indeed, when co-incubated with enterobacterial or meningococcal LPS, CyP induced a dose-dependent reduction of pro-inflammatory cytokine production mediated by an antagonistic interaction with TLR4-MD2 receptor complex [27,28]. CyP was shown to inhibit pro-inflammatory cytokine production also when added some hours after LPS challenge [27]. Furthermore, TNF-α production was demonstrated to be post-transcriptionally regulated [27], thus suggesting a more complex mechanism of action exerted by CyP. In this work, we analyzed the response of human monocytic THP-1 cells to Pg-LPS, and studied the effects of CyP in co-incubation experiments or by adding CyP after Pg-LPS triggering. We measured TNF-α, IL-1β, IL-8 productions and the expression of miR-146a and miR-155. Cultures employing *E. coli* LPS (Ec-LPS) as the trigger of the pro-inflammatory response were used for comparisons. Results demonstrated that CyP inhibits TNF-α, IL-1β and IL-8 productions, also when added two hours after Pg-LPS, with a similar behavior to that observed when using Ec-LPS. Analysis of miR-146a and miR-155 expression showed different effects of CyP treatment in cultures stimulated with Pg-LPS in comparison with Ec-LPS. Although CyP is a LPS antagonist, no decreases of miR-146a expression in cell cultures with CyP added were found. No effect was found on miR-155 expression by CyP treatment in cell cultures stimulated with Pg-LPS, whereas inhibition was observed when co-incubated with Ec-LPS.

## 2. Results

### 2.1. CyP Dose-Dependently Inhibits Pro-Inflammatory Cytokine Production Induced by Pg-LPS

**Figure 1:**
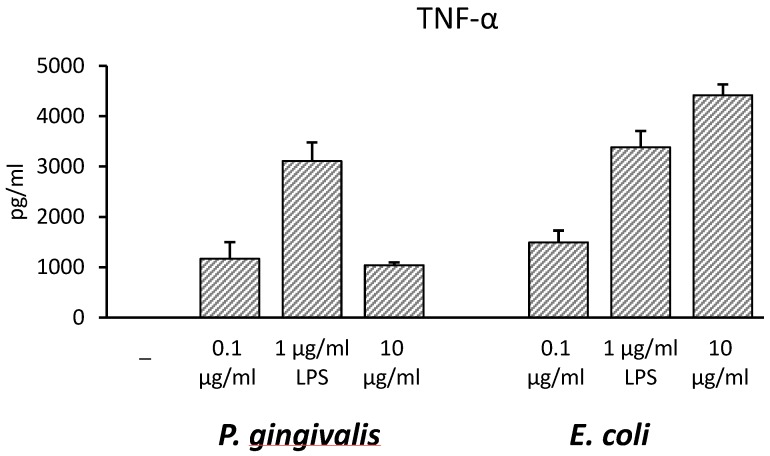
Effects of increasing concentrations of lipopolysaccharide (LPS) on TNF-α released in culture supernatants of THP-1 cells. THP-1 cells were stimulated with *P. gingivalis* (Pg-LPS) or *E. coli* LPS (Ec-LPS) (0.1, 1, 10 µg/mL) for 16–18 h, then supernatants were collected and TNF-α measured by ELISA. The results are shown as mean ± SD of triplicate data.

THP-1 monocytic cells were incubated with Pg-LPS or Ec-LPS at different concentrations (0.1, 1, 10 µg/mL) for 16–18 h with the aim of finding the optimal LPS concentration to induce pro-inflammatory cytokine production. TNF-α was chosen as a reference cytokine and results, demonstrated that 1 µg/mL was the optimal dose to induce the maximal cytokine amount by Pg-LPS (Figure 1). 

**Figure 2:**
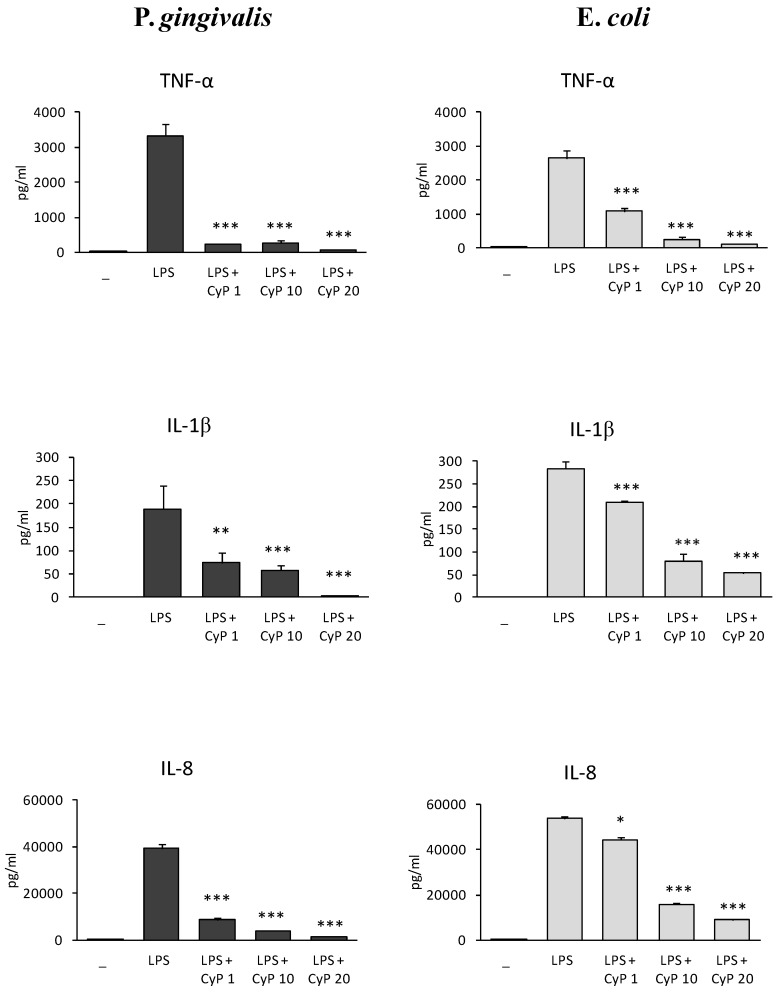
Dose-dependent inhibition of pro-inflammatory cytokine production by CyP. THP-1 cells were stimulated with Pg-LPS or Ec-LPS (1 µg/mL) with CyP added at concentrations of (1, 10, 20 µg/mL) for 16–18 h. Supernatants were collected and TNF-α, IL-1β and IL-8 measured by ELISA. The results are shown as mean ± SD of triplicate data. * *p* < 0.05; ** *p* < 0.01; *** *p* < 0.001.

Based on this result, further experiments were performed co-incubating LPS 1 µg/mL with CyP at different doses (1, 10, 20 µg/mL). The production of TNF-α, IL-1β and IL-8 was studied in cell culture supernatants (Figure 2). Results showed that CyP inhibited very efficiently and dose-dependently pro-inflammatory cytokine production in cultures stimulated with Pg-LPS. To exclude that the downregulation of pro-inflammatory cytokine production by CyP was due to cytotoxicity, cell viability was assessed by trypan blue dye exclusion test both in co-incubation experiments and in cultures incubated with CyP alone. Results showed cell viability >95% in every experimental session. In control cultures incubated with CyP alone no induction of TNF-α, IL-1β, and IL-8 was observed. At a concentration of 1 µg/mL, CyP inhibited the secretion of TNF-α induced by Pg-LPS by 93%, the secretion of IL-1β by 61%, and IL-8 by 78%, respectively. Cultures stimulated with Ec-LPS confirmed a dose-dependent inhibition by CyP; remarkably, inhibitions by CyP at a concentration of 1 µg/mL were milder in Ec-LPS-stimulated cultures than in cultures stimulated with Pg-LPS. Mean inhibitions of TNF-α, IL-1β, and IL8 were 59%, 26%, and 18%, respectively. Independently of LPS source, CyP at a concentration of 20 µg/mL was shown to almost completely inhibit pro-inflammatory cytokine production both in Pg-LPS-stimulated cultures (>90% inhibitions) and Ec-LPS-stimulated ones (>80% inhibitions) (Figure 2).

### 2.2. CyP Inhibits Pro-Inflammatory Cytokine Production Induced by Pg-LPS also When Added Two Hours after LPS

**Figure 3:**
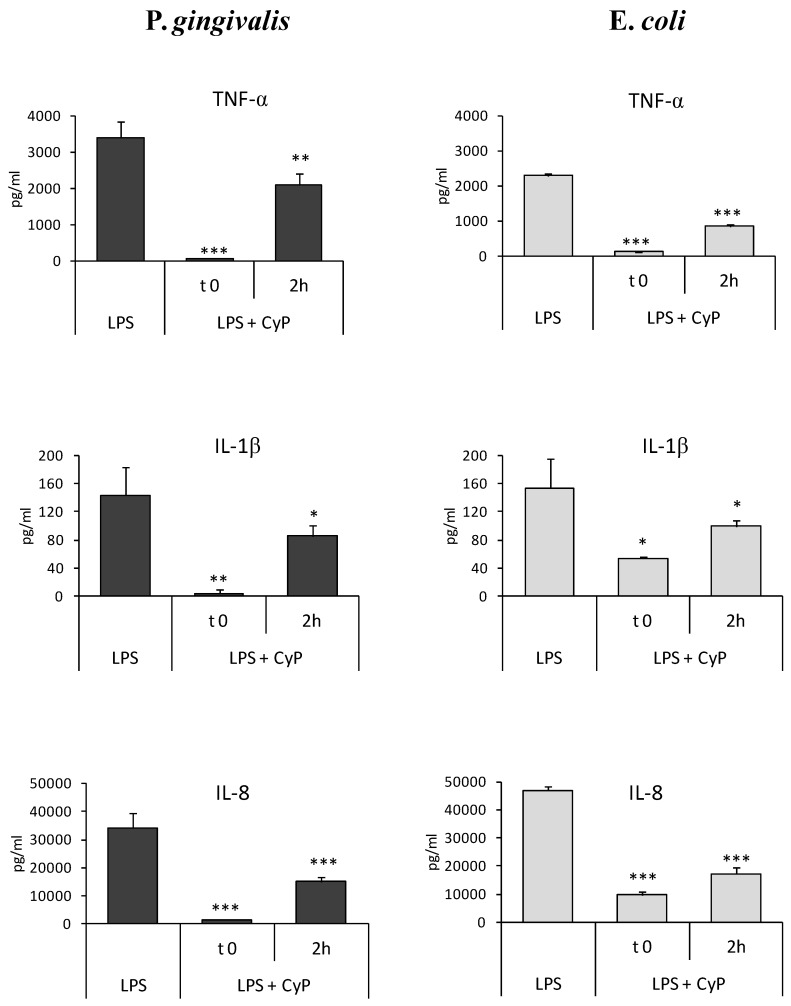
Inhibition of pro-inflammatory cytokine production by CyP co-incubated or added 2 h after Pg-LPS or Ec-LPS. THP-1 cells were stimulated with Pg-LPS or Ec-LPS (1 µg/mL) with CyP (20 µg/mL) added together with LPS (t 0) or 2 h after LPS challenge (2 h). Cells were incubated for 16-18 h, then supernatants were collected and TNF-α, IL-1β and IL-8 measured by ELISA. The results are shown as mean ± SD of triplicate data. * *p* < 0.05; ** *p* < 0.01; *** *p* < 0.001.

The experiments were done using CyP at a concentration of 20 µg/mL in order to study whether CyP was able to inhibit pro-inflammatory cytokine production when added some hours after Pg-LPS. The amounts of cytokines produced in cell culture supernatants were measured after 16–18 h of culture. Results showed that CyP was able to downregulate pro-inflammatory cytokine production, in human monocytic THP-1 cells, even when added in culture 2 h after Pg-LPS triggering (Figure 3). Mean inhibition ranged from 38% for TNF-α to 56% for IL-8 secretion. Results were similar to those obtained with Ec-LPS, where inhibition by CyP ranged from 35% for IL-1β to 62% for TNF-α secretion (Figure 3). 

**Figure 4:**
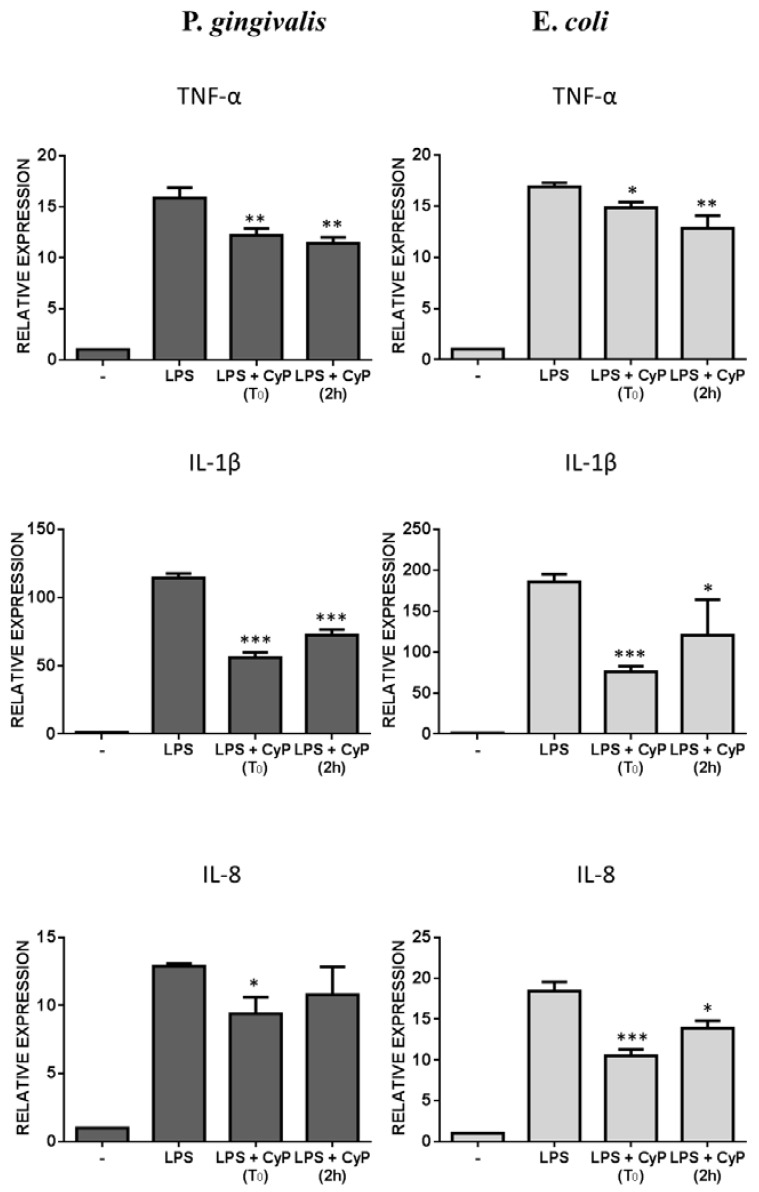
Effects of CyP on pro-inflammatory cytokine mRNA expression. THP-1 cells were stimulated with Pg-LPS or Ec-LPS (1 µg/mL) with CyP (20 µg/mL) added together with LPS (t 0) or 2 h after LPS challenge (2 h). Cells were incubated for 4 h, then were collected and total RNA extracted. mRNA expression was analyzed by RT-PCR and given as relative expression over the mRNA level expressed by untreated cells. The results are shown as mean ± SD of triplicate data. * *p* < 0.05; ** *p* < 0.01; *** *p* < 0.001.

Analysis of mRNA expressions confirmed the results obtained measuring cytokine release in cell culture supernatants (Figure 4). Since LPS treatment induced a peak of mRNA expression for TNF-α within 4 h in human monocyte-derived dendritic cells [27], we evaluated pro-inflammatory cytokine mRNA expressions after 4 h of culture. Results, reported in Figure 4, showed a reduction of TNF-α and IL-1β mRNA expressions in cultures stimulated with Pg-LPS and CyP. For IL-8 a decrease of IL-8 mRNA was observed only after co-incubation of CyP and Pg-LPS. Control cultures employing Ec-LPS showed reductions of all pro-inflammatory cytokines (TNF-α, IL-1β, and IL-8) either in experiment of co-incubation or in experiments where CyP was added after Ec-LPS (Figure 4).

### 2.3. CyP Differently Affects miRNA Expression in THP-1 Cells Stimulated with Pg-LPS and Ec-LPS

**Figure 5:**
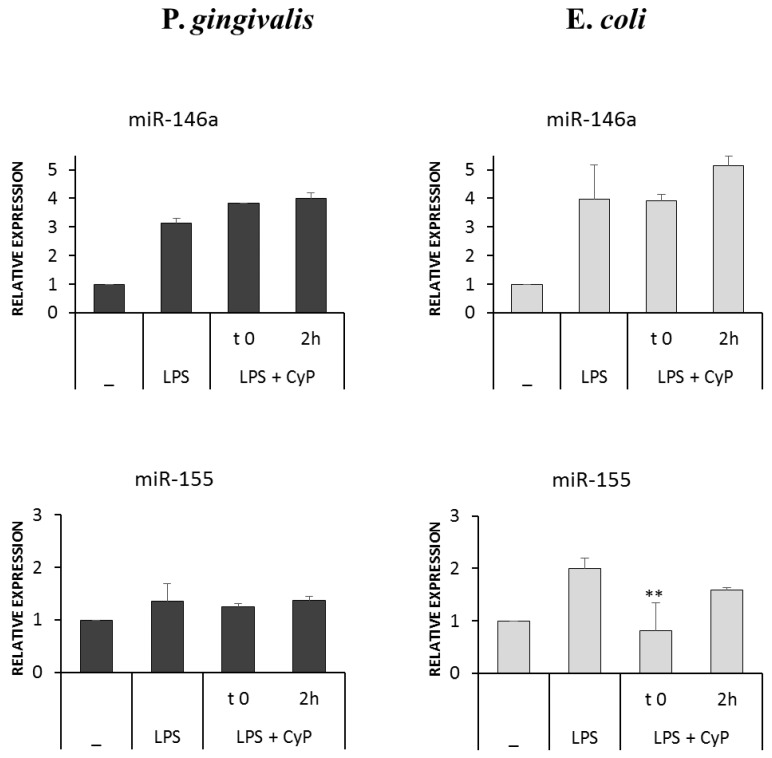
Effects of CyP on miR-146 and miR-155 expressions. THP-1 cells were stimulated with Pg-LPS or Ec-LPS (1 µg/mL) with CyP (20 µg/mL) added together with LPS (t 0) or 2 h after LPS challenge (2 h). Cells were incubated for 4 h, then were collected and total RNA extracted. miRNA expression was analyzed by RT-PCR and given as relative expression over the miRNA level expressed by untreated cells. The results are shown as mean ± SD of triplicate data. ** *p* < 0.01.

CyP being an LPS antagonist acting at the level of TLR4-MD2 receptor complex, we wondered whether CyP might influence miRNA induced by LPS treatment. MiR-146a and miR-155 expressions were studied after 4 h of culture. Results, reported in Figure 5, showed that CyP did not inhibit miR-146a expression in cultures stimulated either with Pg-LPS or with Ec-LPS. Furthermore, CyP did not affect miR-155 expression in cultures with Pg-LPS, whereas inhibited miR-155 expression when co-incubated with Ec-LPS.

## 3. Discussion

In this study, we analyzed the effects of CyP, a cyanobacterial LPS with TLR4-MD2 antagonist activity, on human monocytic THP-1 cells stimulated with Pg-LPS. *P. gingivalis* is a periodontopathic pathogen able to elicit a strong pro-inflammatory response in gingival fibroblasts, endothelial cells and in host immune cells [1,7]. It has been demonstrated that *P. gingivalis* can produce heterogeneous LPS structures characterized by diversified capability of inducing IL-6 and IL-8 productions in human gingival fibroblasts [10,17]. Our results confirmed that commercially available LPS preparations from *P. gingivalis*, which contain mainly penta-acylated lipid A structures, as demonstrated by Nativel and colleagues [15], can induce high releases of TNF-α, IL-1β, and IL-8 by human monocytic THP-1 cells. In our experiments, the amounts of TNF-α and IL-1β induced by Pg-LPS were similar to those observed in Ec-LPS-stimulated cultures; differently, the levels of IL-8, evaluated both as cytokine produced in cell culture supernatants and as mRNA expression, resulted lower in cultures stimulated with Pg-LPS compared with those stimulated with Ec-LPS. This result on human monocytic cells is in line with previous observations obtained on human gingival fibroblasts and neutrophils on IL-8 production [1,29], thus confirming how structural variations of lipid A in LPS structures influence cell response. Remarkably, cyanobacterial LPS antagonist, CyP, was shown to inhibit with high efficiency pro-inflammatory cytokine production in Pg-LPS-stimulated cultures (Figure 2). CyP effectiveness in the inhibition of the pro-inflammatory response was already high at 1 µg/mL, and was almost complete at a concentration of 20 µg/ml. Previous studies demonstrated that CyP was able to downregulate pro-inflammatory cytokine production induced by enterobacterial or meningococcal LPS through an antagonistic interaction with TLR4-MD2 receptor complex [27,28]. Indeed, although the levels of TNF-α and IL-6 released by monocyte-derived dendritic cells stimulated with enterobacterial or meningococcal LPS were similar, the amounts of CyP needed for the inhibition of TNF-α and IL-6 were higher in cultures stimulated with meningococcal LPS compared with the Ec-LPS-stimulated ones. In our experiments, we found higher efficacy of CyP in inhibiting TNF-α, IL-1β, and IL-8 in cultures stimulated with Pg-LPS than in those stimulated with Ec-LPS. These results suggest that CyP can block the interaction between TLR4-MD2 complex and LPS with different efficiency, depending on the relative affinity between the receptor complex and each LPS structure.

Previously published results showed that CyP is able to inhibit TNF-α, IL-6, and IL-12 also when added some hours after Ec-LPS in human monocyte-derived dendritic cells [27]. In this study, using human THP-1 cells, we confirmed the inhibitory activity on TNF-α, and we showed a significant downregulation of IL-1β and IL-8 productions, not only in cultures stimulated with Ec-LPS, but also in cultures performed in the presence of Pg-LPS (Figure 3). The capability of CyP to inhibit pro-inflammatory cytokine production was also confirmed at the mRNA level.

Recent results demonstrated that *P. gingivalis* infection activates complex epigenetic regulations involving miRNA [18,30,31,32]. LPS represents one of the virulence factors influencing miRNA expression [22,33,34]. In particular, it was confirmed that miR146a represents a negative regulator induced by LPS to control pro-inflammatory response, having a direct role in the downregulation of TNF-α production [19,20,34]. Differently from gram-negative LPS, we recently demonstrated that incubation of human monocytes with CyP alone, specifically triggers miR-146 expression, while having no effects on miR-155 or on pro-inflammatory cytokine induction [35]. Based on this result, we evaluated the effects of CyP added in cultures together with Pg-LPS or Ec-LPS on miR-146a and miR-155 expressions. Our results showed that miR-146a is induced early by Pg-LPS and Ec-LPS, whereas this was not observed for miR-155 expression in the cell cultures incubated with Pg-LPS. At present, we do not know whether this difference in the timing of the induction of miR-146a and miR-155 by Pg-LPS could be relevant in the regulation of host immune response to *P. gingivalis* infection. Further time course experiments using human primary monocytes are needed to fully exploit this possibility and to study the effect of CyP since these could add new information about the mechanisms regulating the pro-inflammatory response induced by Pg-LPS. 

## 4. Conclusions

In this study, we highlighted some mechanisms concerning the regulation of the pro-inflammatory response induced by Pg-LPS. By means of CyP, a cyanobacterial LPS structure with TLR4-MD2 antagonist activity, we showed that pro-inflammatory activation induced by Pg-LPS can be efficiently downregulated by CyP, also some hours after Pg-LPS challenge. TNF-α, IL-1β and IL-8 inhibitions resulted significant, also because the expression of the negative regulator of the pro-inflammatory response, e.g., miR-146a, was preserved by CyP treatment. The possibility of limiting pro-inflammatory cytokine response after disease onset, by anti-inflammatory molecules targeting TLR4-MD2, could represent a useful adjunct for the treatment of periodontal disease, in which it is important to eradicate infection but also to stem the negative effects of the LPS released by periodontopathic bacteria.

## 5. Materials and Methods

### 5.1. Cells and Reagents

Human THP-1 monocytic cell line was obtained from Istituto Zooprofilattico Sperimentale della Lombardia e dell’Emilia (Brescia, Italy). Cells were maintained by twice weekly passage in complete RPMI 1640 medium (Euroclone, Milano, Italy), containing 10% fetal bovine serum (FBS, Gibco, Grand Island, NY, USA), 2 mM l-glutamine (Euroclone, Milano, Italy), 50 U/mL penicillin, 50 µg/mL streptomycin (Euroclone, Milano, Italy), 0.05 mM 2-mercaptoethanol. Cells were incubated at 37 °C in 5% CO_2_ and cultures were performed at a density of 5 × 10^5^ cells/mL in all experiments.

Ultrapure LPS from *Porphyromonas gingivalis* (Pg-LPS) was purchased by Invivogen (San Diego, CA, USA), ultrapure LPS from *E. coli* (Ec-LPS, serotype O111:B4) was purchased by Sigma Aldrich (St. Louis, MO, USA), and dissolved in sterile endotoxin-free phosphate-buffered saline (PBS) at a concentration of 1 mg/mL. Further dilutions were made in complete medium. LPS was visualized by electrophoresis in SDS-PAGE (Precast gel, Invitrogen, Carlsbad, CA, USA) and silver staining after periodate oxidation (PlusOne silver staining kit, GE Healthcare, Uppsala, Sweden) (Figure S1). 

Cyanobacterial LPS antagonist (CyP) was extracted from the freshwater cyanobacterium *Oscillatoria planktothrix FP1* by a phenol-guanidinium thiocyanate-based method, as described elsewhere [27]. The product was treated with DNase (20 µg/mL) and RNase (10 µg/mL) in 50 mM TRIS buffer, pH 7.5 containing 10 mM MgCl_2_, for 2 h at room temperature prior to addition of proteinase K (100 µg/mL) for an overnight incubation at 37 °C. The sample was then re-extracted, purified by ion exchange chromatography with Sartobind Q (Sartorius Stedim Biotech, Goettingen, Germany), centrifuged in Zeba spin desalting columns (Pierce, Rockford, IL, USA), and freeze-dried. The final product was analyzed by size exclusion chromatography in HPLC, using evaporative light scattering detector (column Superose 12; GE healthcare Little Chalfont, UK; HPLC, Agilent 1200), visualized by electrophoresis in SDS-PAGE and silver staining after periodate oxidation (Figure S1). Purity of CyP was >90%. Protein contamination was <2% (Bradford method); nucleic acid contamination measured spectrophotometrically (260–280 nm) was 0.5%. CyP preparations contained <1 Endotoxin units/µg measured by endpoint chromogenic LAL test (Lonza Group, Basel, Switzerland). CyP was dissolved in sterile, endotoxin-free PBS prior to addition to cell cultures.

### 5.2. THP-1 Cell Cultures

Cells at the concentration of 5 × 10^5^ cells/mL were seeded in sterile 6-well plates, and incubated with or without Pg-LPS and Ec-LPS at different concentrations (0,1-1-10 µg/mL) for 16–18 h at 37 °C in 5% CO_2_ to assess the optimal concentration of LPS to be employed for cytokine production. Further cultures were performed using Pg-LPS or Ec-LPS 1 µg/mL with or without CyP at the final concentration of 1, 10, 20 µg/mL. In some experimental sessions, CyP, at a concentration of 20 µg/mL, was added together with LPS and 2 h after LPS. Control cultures employing CyP alone at 1, 10, 20 µg/mL were also carried out. Supernatants were collected after 16–18 h of incubation at 37 C in 5% CO_2_, centrifuged, and then stored at −80 °C for subsequent cytokine analyses. At the end of all incubations, cell viability was assessed by trypan blue exclusion test. Analyses of cytokine mRNAs and miRNA expression were done on THP-1 cell cultures incubated for 4 h at 37 °C in 5% CO_2_. Cell pellets were collected and stored at −80 °C for RNA extraction. 

### 5.3. Cytokine ELISA

TNF-α, IL-1β, IL-8 were measured in supernatants of cell cultures using enzyme-linked immunosorbent assays (ELISA) kits (Diaclone, Besançon Cedex, France), according to the manufacturer’s instructions, and calibrated with commercial cytokine molecules in the kits. Absorbance was measured at 450 nm with a TECAN microplate reader (Tecan Group Ltd., Mannedorf, Switzerland); standard curves were constructed and cytokine concentrations of the samples were read off these curves.

### 5.4. RNA Isolation

RNA isolation was carried out using automated Maxwell instrument and Maxwell 16 LEV simplyRNA cell kit (Promega, Corporation, Madison, WI, USA). The RNA concentration was determined using a NanoDrop spectrophotometer and equal amounts of each RNA were used for real-time PCR (qRT-PCR) analysis.

### 5.5. Cytokine Quantitative Real-Time PCR

Expression of cytokine mRNAs was performed using an Iscript cDNA synthesis kit and an ITAQ Universal Probes Supermix (Bio-Rad Laboratories s.r.l., Milano, Italy). Briefly, 400 ng total RNA was reverse transcribed in a final volume of 20 µL, according to the manufacturer’s instructions. PCR was conducted in a 20 µL volume reaction containing 4 µL 1/5 diluted cDNA template, and PrimePCR^TM^ probe assays (TNF-α unique assay ID qHsaCEP0040184; IL-1β qHsaCIP033362; IL-8 qHsaCEP0053894) labeled with a FAM fluorophore (Bio-Rad Laboratories s.r.l., Milano, Italy). Relative expressions were calculated compared with unstimulated controls, after normalizing the change in expression of the gene of interest to the housekeeping gene β-actin (unique assay ID qHsaCEP0036280), using the ΔΔ*C*_t_ method [36].

### 5.6. MiRNA Quantitative Real-Time PCR

MiRNA analyses were performed using miRCURY LNA^TM^ Universal RT microRNA PCR kit (Exiqon A/S, Vedbaek, Denmark). Briefly, 50 ng of RNA were reverse transcribed in 10 µL final volume, according to the manufacturer’s instructions. Quantitative real-time PCRs were performed in final volumes of 10 µL reaction containing 4 µL 1/25 diluted cDNA template, using SYBR green and LNA^TM^ enhanced primers (targets: hsa-miR-146a-5p, hsa-miR-155-5p) designed by Exiqon for optimal performance with miRCURY LNA^TM^ Universal cDNA Synthesis kit II and ExiLENT SYBR. Melting curves were done to confirm that any product was specific to the desired amplicon. Stably expressed miR-103a-3p was employed as reference gene and used for normalizing target expressions. 

### 5.7. Statistical Analysis

One-way ANOVA with Dunnet Multiple Comparison post hoc test was performed to assess statistical significance (*p* values < 0.05) using GraphPad Prism 6.0 (GraphPad software Inc., San Diego, CA, USA).

## Supplementary Materials

The following are available online at http://www.mdpi.com/2072-6651/10/7/290/s1, Figure S1, SDS-PAGE electrophoresis of LPS and CyP.
