# Supplementary material for: The Effect of Cyanobacterial LPS Antagonist (CyP) on Cytokines and Micro-RNA Expression Induced by Porphyromonas gingivalis LPS
Source: Toxins (Basel). 2018 Jul 16;10(7):290. doi: 10.3390/toxins10070290 (PMC6071223; doi:10.3390/toxins10070290)
Supplement: Supplementary file 1 [file toxins-10-00290-s001.pdf]

Supplementary Materials: The Effect of Cyanobacterial LPS Antagonist (CyP) on Cytokines and Micro-RNA Expression Induced by *Porphyromonas gingivalis* LPS

Monica Molteni, Annalisa Bosi, and Carlo Rossetti

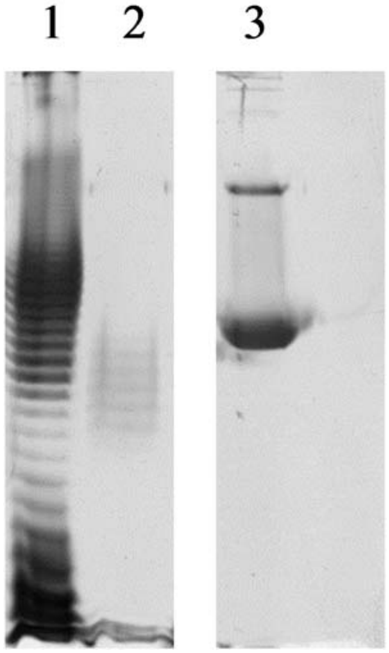

Figure S1. SDS-PAGE electrophoresis of LPS and CyP. Ec-LPS, Pg-LPS and CyP were visualized by electrophoresis in SDS-PAGE and silver staining after periodate oxidation. Lane 1: Ec-LPS (serotype O111:B4) 16 μg; lane 2: Pg-LPS 16 μg; lane 3: CyP 8 μg.
